# Nanofibrous Membrane-Based Stretchable Electrochemical Sweat Sensor for pH Detection

**DOI:** 10.3390/polym17050663

**Published:** 2025-02-28

**Authors:** Longzhou Zhang, Baoyuan Ma, Zhiguang Xu, Yan Zhao

**Affiliations:** 1 College of Textile and Clothing Engineering, Soochow University, Suzhou 215123, China; 2 College of Biological, Chemical Sciences and Engineering, Jiaxing University, Jiaxing 314001, China

**Keywords:** wearable sweat sensor, pH detection, electrospun membrane, stretchable conductive electrode

## Abstract

Wearable, non-invasive sweat sensors capable of continuously monitoring the pH of sweat, which is a key indicator related to metabolism and homeostasis level, are highly desirable for personal health management. However, ensuring the stability and accuracy of these sensors can be challenging when the body is in motion. In this work, we prepared a stretchable nanofibrous membrane-based electrochemical pH-sensing electrode by embedding carbon nanotubes (MWCNT) and silver nanowires (AgNWs) into an elastic electrospun nanofibrous membrane, followed by polyaniline electrodeposition. The as-prepared pH-sensing electrode showed a high sensitivity of 82.53 mV/pH and high accuracy in ionic solutions with pH ranging from 3 to 7. Notably, the electrode maintained stable sensing performance under deformations, including torsion, bending, and tensile strains up to 30%. Even after 1000 cycles of stretching at a 30% tensile strain, the detection sensitivity remained above 70 mV/pH, indicating its potential application as a wearable electrochemical sensor for monitoring sweat pH in personal health management.

## 1. Introduction

Wearable electrochemical sensors have been rapidly developed and drawing significant attention in the field of real-time, continuous health monitoring [1,2,3] due to their high sensitivity, accuracy, fast response, and compact size [4,5,6]. Sweat has become a key target for non-invasive detection [7] as it contains various health-related biomarkers that offer insights into physiological processes [8,9,10,11]. Electrochemical analysis of sweat enables health assessment at a molecular level [12,13]. Among the primary indicators in sweat, pH reflects the body’s acid-base balance and metabolic status, aiding in diagnosing renal disorders and skin conditions [14,15]. Furthermore, pH measurement can indicate wound healing progress [16], playing an important role in reflecting overall health [17].

However, applying electrochemical sensors to the skin surface presents several challenges, particularly the need for electrodes to maintain stable performance despite deformation caused by body movements [18]. Current strategies for fabricating stretchable electrochemical electrodes generally fall into two main categories. The first approach involves enhancing the flexibility of rigid materials to effectively disperse mechanical stress during mechanical deformation. The second strategy employs highly conductive nanomaterials and makes them deposit onto elastic substrates. This ensures that the conductive network remains intact under tensile strain, thereby preserving the electrode’s electrical conductivity upon mechanical deformation [19].

Recent studies have explored various solutions to develop electrochemical sensing electrodes that maintain relatively stable sensing performance even under mechanical strain. These approaches aim to enhance the flexibility and durability of the electrodes, ensuring their functionality when subjected to stretching, bending, or deformation. For example, Wang et al. [20] prepared highly elastic stretchable electrodes for pH monitoring by using gold fibers with a pleated surface structure, effectively dispersing mechanical strain and allowing for stable electrochemical detection even when the electrode was stretched. Kil et al. [21] integrated a stretchable graphene electrode onto a designed sock, by printing a well-conducting graphene ink onto a serpentine structure of degradable elastic plastics. Such a stretchable graphene electrode enables the electrochemical detection of sodium ion when it is under diverse mechanical deformations. Hua et al. [22] established a three-dimensional composite of graphene and carbon nanotube frameworks on the surface of a carbon electrode, through the electrochemical reduction on an elastomeric substrate. The resulting NH_4_^+^ sensor based on this electrode was able to maintain stable detection performance when the electrode was under tensile strains from 10% to 40%. Zhai et al. [23] reported a stretchable gold mushroom-shaped electrode that can be directly grown on an elastomeric substrate, with nanoparticles serving as the “head” and nanowires as the “tail”. The three-dimensional vertical nanowires provide a high surface area and excellent electrical conductivity for electrochemical reactions. The modified glucose sensing electrode can maintain high sensing performance under 30% strain and can also effectively detect tensile strains. Despite the accuracy and flexibility of existing stretchable electrochemical sensing electrodes, many fabrication processes remain complex or rely on costly conductive materials, presenting challenges for large-scale production and practical applications.

In this work, we prepared a flexible, stretchable electrochemical pH-sensing electrode by using a highly elastic electrospun TPU nanofiber membrane as substrate. The structure of the electrospun nanofibrous membranes, with their high surface area and interconnected pores, provides an ideal framework for the incorporation of conductive nanomaterials such as silver nanowires (AgNWs) and multi-walled carbon nanotubes (MWCNT). Loading these conductive nanomaterials within the nanofibrous membrane resulted in a continuous and stable conductive network, thus ensuring both high conductivity and stability in the conductivity when being stretched. After electrochemical deposition of polyaniline (PANI) on the highly conductive membrane, a pH-sensing electrode was obtained, and the as-obtained pH-sensing electrode exhibited a high sensitivity of up to 82.53 mV/pH in pH-sensing tests. Furthermore, the pH-sensing electrode maintained stable and superior sensing performance under torsion, bending, and even 30% tensile strain, highlighting its great potential for applications in wearable sweat detection and monitoring.

## 2. Materials and Methods

### 2.1. Materials

Polyurethane (TPU) was purchased from Dongguan Derun Plastic Chemical Co, Dongguan, China. *N*, *N*-Dimethylformamide (DMF) was obtained from Utep Technology Suzhou Co., Ltd., Suzhou, China. Multi-walled carbon nanotube (MWCNT) aqueous paste was purchased from Suzhou Carbonfontaine Graphene Science & Technology Co., Ltd., Suzhou, China. Silver nitrate (AgNO_3_) was purchased from Shanghai Qiangxun Chemical Reagent Co., Ltd., Shanghai, China, and ferric chloride (FeCl_3_) was obtained from Shanghai Bailing Wei Chemical Technology Co., Ltd., Shanghai, China. Potassium ferricyanide (K_3_[Fe(CN)_6_]) was acquired from Anhui Senrise Technology Co., Ltd., Anqing, China. Aniline (ANI), sodium bicarbonate (NaHCO_3_), and polyvinylpyrrolidone (PVP) were purchased from Shanghai Aladdin Biochemical Science and Technology Co., Ltd., Shanghai, China. Polyvinyl chloride, sodium chloride (NaCl), potassium chloride (KCl), and calcium chloride (CaCl_2_) were purchased from Shanghai Lingfeng Chemical Reagent Co., Ltd, Shanghai, China. Sulfuric acid (H_2_SO_4_), hydrochloric acid (HCl), anhydrous ethanol, and ethylene glycol (EG) were obtained from Jiangsu Qiangsheng Functional Chemical Co., Ltd., Changshu, China.

### 2.2. Preparation of TPU Electrospun Membrane

TPU spinning solution with a concentration of 16% was prepared by adding TPU into DMF, followed by stirring at room temperature for 10 h with ultrasonication for 30 min every 2.5 h until the TPU was completely dissolved to form a homogeneous solution. The solution was then transferred to a 20 mL syringe for electrospinning. The parameters of the electrospinning were as follows: the distance between the syringe tip and the receiving roller was 15 cm, the flow rate of syringe pump was 1.0 mL/h, the spinning voltage ranged from 15 to 20 kV, the temperature was maintained at 25 °C, the relative humidity was 40%, the round-trip distance of the needle was 100 mm, and the needle speed was 5 mm/s. After 8 h spinning, the TPU electrospun membrane (TPUEM) was peeled off from the receiving roller and dried at room temperature.

### 2.3. Preparation of Silver Nanowires

The ethylene glycol (EG) was distilled at 130 °C for 12 h. After cooling to room temperature, 2.5 g of PVP was added to 40 mL of the prepared EG and stirred until completely dissolved. Separately, another solution was prepared by adding 0.02 g of FeCl_3_ and 0.85 g of AgNO_3_ into 20 mL of EG, followed by stirring. The two solutions were then mixed and stirred for 15 min. The obtained solution was slowly heated to 150 °C for 10–20 min and maintained at this temperature for 5 h before being cooled to room temperature. The obtained silver nanowires (AgNWs) were then centrifuged in acetone at 5000 rpm for 10 min and washed with ethanol to remove small particles such as FeCl_3_ and AgNO_3_. The centrifugation and washing steps were repeated three times.

### 2.4. Preparation of MWCNT Dispersion

MWCNT aqueous paste was diluted with water to prepare a 5 wt% aqueous dispersion of MWCNT. To ensure effective dispersion, the aqueous dispersion solution was ultrasonicated for 30 min and then stirred for an additional 30 min.

### 2.5. Preparation of TPU Membrane Loaded with AgNWs

Anhydrous ethanol was used to prepare the AgNWs dispersion at a concentration of 10 mg/mL. After stirring, the obtained TPUEM was cut into rectangular fiber membranes (30 mm × 10 mm) and subsequently immersed in the AgNWs dispersion for 20 min. During this impregnation process, the TPUEM membrane was repeatedly stretched and released to make the AgNWs easily diffuse into the internal pores among nanofibers. After immersion, the loosely attached AgNWs on the surface of TPUEM were washed away with ethanol, and then the obtained TPUEM/AgNWs membrane was dried in a vacuum oven at 50 °C for 60 min.

### 2.6. Preparation of TPU Membrane Loaded with AgNWs and MWCNT

The TPUEM/AgNWs membrane was then immersed in the 5 wt% MWCNT aqueous dispersion for further loading. After 15 min immersion with simultaneous ultrasonic treatment, the membrane was taken out, and the loosely attached MWCNT on the membrane surface was rinsed away with deionized water, and then dried in an oven at 60 °C for 40 min to obtain the TPUEM/AgNWs/MWCNT membrane.

### 2.7. Preparation of pH-Sensing Electrode

The pH-sensing electrode was prepared by using the as-prepared highly conductive TPUEM/AgNWs/MWCNT membrane as the conductive substrate, and modifying it with PANI via electrochemical deposition method. The electrochemical deposition was conducted in a three-electrode system. Specifically, the TPUEM/AgNWs/MWCNT conductive membrane was used as the working electrode (WE), together with commercially available reference (RE) and counter electrodes (CE). Before electrochemical deposition, the surface of the TPUEM/AgNWs/MWCNT membrane was cleaned using cyclic voltammetry with a scan rate of 40 mV/s over a voltage range of −0.5 V to 1 V in a 0.5 mol/L HCl solution. The cleaning process involved 15 cycles, after which the conductive membrane was washed and dried at room temperature. Subsequently, polyaniline was electrochemically deposited onto the surface of the TPUEM/AgNWs/MWCNT conductive membrane via cyclic voltammetry, in an electrolyte solution containing 0.1 mol/L aniline and 0.01 mol/L sulfuric acid, with 30 cycles at a voltage range of −0.2 V to 1 V and a scan rate of 20 mV/s. The polyaniline deposition was performed in two stages: the first 15 cycles were completed with the initial electrolyte, followed by an additional 15 cycles with fresh electrolyte.

### 2.8. Characterizations

The surface morphology of electrospun membranes was observed using a high-resolution field emission scanning electron microscope (SEM, Regulus 8100, Hitachi, Tokyo, Japan). The electrical resistance of the TPUEM/AgNWs/MWCNT membrane was measured with a benchtop digital multimeter (Keithley DMM, Tektronix, Beaverton, OR, USA), and the resistivity was determined using a multifunctional digital four-probe tester (ST-2258C, Suzhou Jingle Electronics Co., Ltd., Suzhou, China), with sample dimension of 30 mm length, 3 mm width, and 300 µm thickness. The X-ray diffraction (XRD) of the samples was conducted with a D8 Advance X-ray diffractometer. Thermogravimetric (TG) analysis was conducted using a DTA 6300 (Seiko Instruments Inc., Chiba, Japan) analyzer under a nitrogen atmosphere up to 600 °C. The sensing performance of pH-sensing electrodes (30 mm in length, 3 mm in width, and 300 µm in thickness) was evaluated using an electrochemical workstation (SP-300, Bio-Logic, Grenoble, France) in the analyte solutions. Energy dispersive X-ray spectroscopy (EDS) was performed using an SEM equipped with ZEISS Sigma 300 (Carl Zeiss AG, Oberkochen, Germany).

## 3. Results and Discussion

### 3.1. Fabrication of the Stretchable Electrode

**Figure 1:**
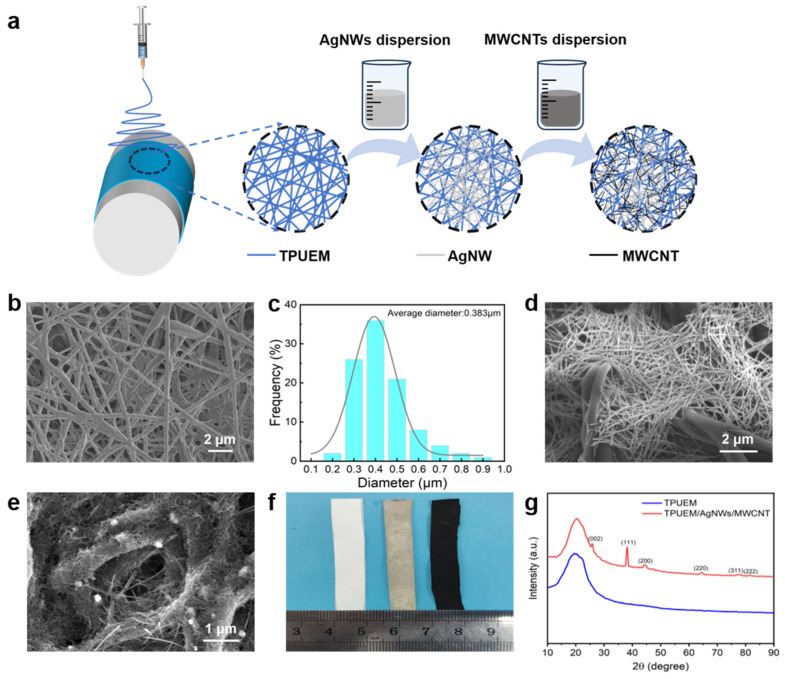
(**a**) Preparation process of the highly conductive TPUEM/AgNWs/MWCNT membrane. (**b**) SEM image and (**c**) fiber diameter for the pristine TPUEM. SEM images of (**d**) TPUEM/AgNWs and (**e**) TPUEM/AgNWs/MWCNT membranes. (**f**) Digital photo of TPUEM (**left**), TPUEM/AgNWs (**middle**), and TPUEM/AgNWs/MWCNT (**right**) membranes. (**g**) XRD patterns of TPUEM and TPUEM/AgNWs/MWCNT membranes.

The experimental process is illustrated in Figure 1a. Specifically, TPU was used as the raw material to fabricate a uniform, highly stretchable nanofibrous membrane (TPUEM) via electrospinning. The TPUEM was then immersed in the ethanol dispersion of AgNWs (Figure S1) and subjected to ultrasonication. During the impregnation process, the TPUEM was repeatedly stretched and released to allow the AgNWs to sufficiently fill the voids within the TPUEM. As shown in Figure 1b, the electrospun fibers in the TPUEM are randomly distributed, with diameters ranging from 300 nm to 500 nm (Figure 1c). After the impregnation with AgNWs, as depicted in Figure 1d, a significant amount of AgNWs were observed in the internal spaces of TPUEM, imparting electrical conductivity to the TPUEM. To further enhance the conductivity and increase the specific surface area for electrochemical sensing, the TPUEM/AgNWs membrane was subsequently treated with an MWCNT dispersion in the same manner as for AgNWs. As shown in Figure 1e, after the treatment, an amount of MWCNT appeared on the surface of TPUEM/AgNWs. The EDS mapping results indicate that AgNWs are uniformly distributed across the surface of TPUEM/AgNWs/MWCNT (Figure S2), further confirming the role of AgNWs in the formation of a continuous conductive network within the TPUEM fibrous structure. From the EDS spectrum, the weight percentage of the Ag element was calculated to be about 6%. The digital photo shown in Figure 1f indicates the color change after the impregnation with AgNWs and MWCNT. It can be seen that, after the sequential loading of AgNWs and MWCNT, the TPUEM changes from pure white to grayish-white, and eventually to black. To further verify the successful incorporation of AgNWs and MWCNT into TPUEM, XRD analysis was conducted on TPUEM before and after impregnation (Figure 1g). The main diffraction peak of TPU is visible at 2θ = 19.9° [24,25]. In the XRD pattern of TPUEM/AgNWs/MWCNT, the sharp peak at 2θ = 26.1° corresponds to the (002) crystal facet of MWCNT [26]. The distinct diffraction peak at 2θ = 38°, along with weaker peaks at 2θ = 44.5°, 64.4°, 77.5°, and 81.4°, represent the (111), (200), (220), (311), and (222) crystal facets of AgNWs, respectively [27]. Furthermore, TG analysis was conducted to assess the loading of AgNWs and MWCNT in TPUEM membrane (Figure S3). Based on the residual mass percentages of the three samples, pristine TPUEM, TPUEM/AgNWs, and TPUEM/AgNWs/MWCNT, the calculated loading of AgNWs was 9.43%, while the loading of MWCNT was 3.81%.

### 3.2. Electrical Conductivity

**Figure 2:**
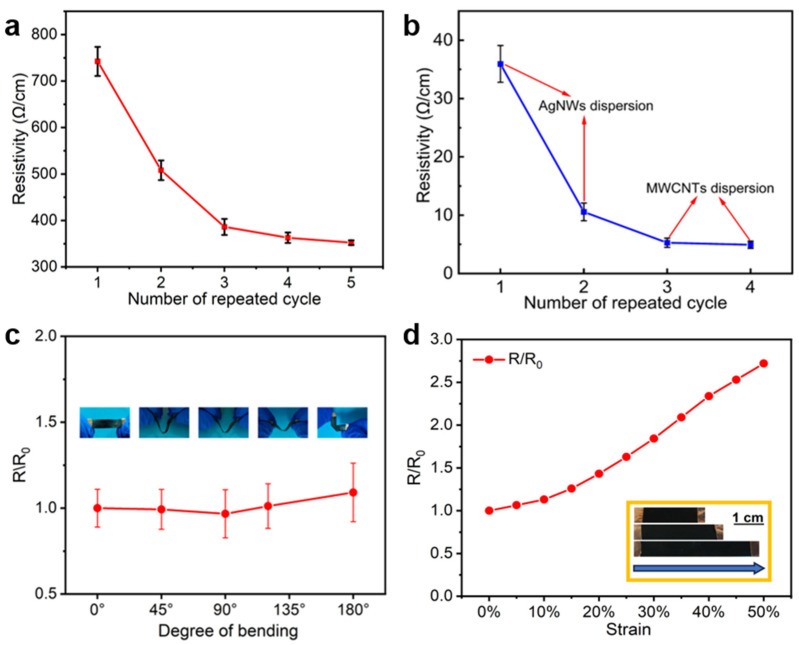
(**a**) Changes in resistivity as impregnation cycles of MWCNT increase. (**b**) Changes in resistivity as impregnation cycles of AgNWs and MWCNT increase. Relative resistance changes of TPUEM/AgNWs/MWCNT electrode under different degrees of (**c**) bending and (**d**) stretching strain.

The resistance changes of TPUEM after impregnation with different amounts of AgNWs and MWCNT were studied. As shown in Figure 2a, the resistance of the TPUEM/MWCNT membrane, which only uses MWCNT as a conductive filler, remained relatively high level (352 Ω/cm) even after five loading cycles. In contrast, the incorporation of AgNWs significantly enhanced the electrode’s conductivity (Figure 2b). After two loading cycles of AgNWs, the electrode resistance of TPUEM reached as low as 10 Ω/cm. Further loading of MWCNT on top of the AgNWs forms a heterogeneous nanocomposite layer, with MWCNT and AgNWs having different length-to-diameter ratios uniformly distributed within the TPUEM (Figure 1e). This nanohybrid layer provides a more continuous and stable conductive pathway than layers of AgNWs or MWCNT alone [28], resulting in a lower resistance (5 Ω/cm) for TPUEM/AgNWs/MWCNT. Further impregnation of MWCNT did not effectively reduce the resistance, which implies that the loading is already enough for constructing an effective conductive network. Notably, the conductivity remained stable under bending (Figure 2c), and the resistance increased only 2.7-fold under 50% stretching strain (Figure 2d). These results indicate that the continuous and stable conductive network formed by AgNWs and MWCNT within the TPUEM not only significantly enhances its electrical conductivity but also leverages the inherent high elasticity and flexibility of TPUEM, yielding a stretchable electrode composite membrane with relatively stable conductivity.

### 3.3. Electrochemical Performance

**Figure 3:**
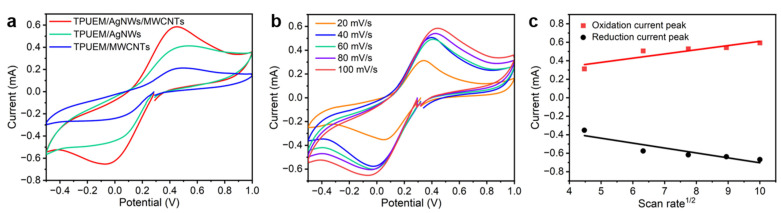
(**a**) CV curves of the three electrodes in a mixed solution of 0.1 M KCl and 5 mM of K_3_[Fe(CN)_6_]. (**b**) CV curves of TPUEM/AgNWs/MWCNT electrode in mixed solutions of KCl and K_3_[Fe(CN)_6_] and (**c**) the linear relationship between the peak currents and the square root of the scan rate.

Cyclic voltammetry (CV) was used to investigate the electrochemical responses of the conductive electrodes of TPUEM/MWCNT, TPUEM/AgNWs, and TPUEM/AgNWs/MWCNT. As shown in Figure 3a, compared to electrodes containing only a single nanomaterial (AgNWs or MWCNT), the TPUEM/AgNWs/MWCNT electrode demonstrated superior electrochemical performance, primarily contributing to the synergistic effect between the two electronic intermediates when MWCNT was added to the space of the underlying AgNWs [1]. Figure 3b shows the electrochemical response of TPUEM/AgNWs/MWCNT at different scanning rates. It can be seen that the peak anodic and peak cathodic currents increase correspondingly with the increase of scan rate. This behavior suggests quasi-reversible redox processes. Additionally, the fitting curves in Figure 3c indicate that a linear correlation between the peak currents and the square root of the scan rate, demonstrating that this redox process is a diffusion-controlled electrochemical process [29].

### 3.4. Sensing Performance

**Figure 4:**
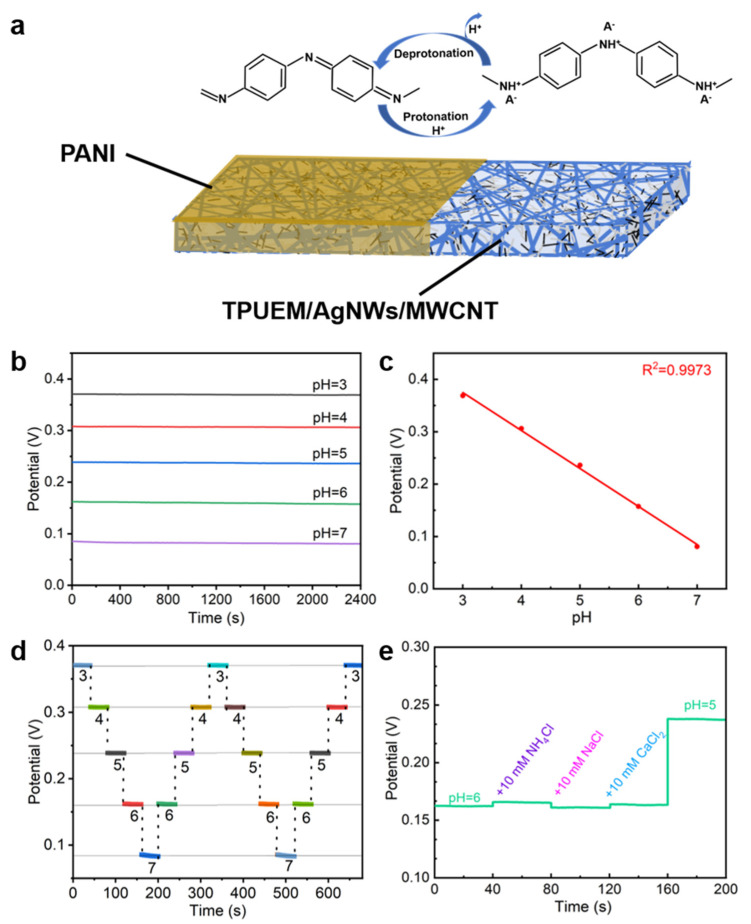
(**a**) Schematic diagram of the working principle of the prepared pH-sensing electrode. (**b**) Potentiometric response of the prepared pH-sensing electrode in electrolyte solution with pH value ranging from 3 to 7 over 40 min and (**c**) the corresponding calibration plot. (**d**) Reversibility test of the prepared pH-sensing electrode. (**e**) Selectivity test of the prepared pH electrode.

A pH-sensing electrode was prepared by electrodepositing PANI onto the surface of TPUEM/AgNWs/MWCNT. From the cyclic voltammetry (CV) curves recorded during the electrodeposition of PANI (Figure S4), it can be seen that, as the number of scan cycles increases, the enclosed area of the CV curves gradually expands, indicating the gradual deposition of PANI onto the surface of TPUEM/AgNWs/MWCNT with increasing the number of scan cycles. Meanwhile, the SEM image of the composite membrane after PANI electrodeposition (Figure S5) clearly reveals that PANI film was formed on the electrode surface. The structure of the as-prepared PANI-based pH-sensing electrode and its pH-sensing mechanism are illustrated in Figure 4a. The fundamental principle of the electrochemical pH sensor is to convert the H^+^ concentration in the solution into an electrical signal. For PANI, this process is primarily realized through changes in conductivity induced by the protonation and deprotonation of PANI on the electrode surface. In an acidic environment, PANI interacts with H^+^ ions to form a doped state with high conductivity, thereby leading to a potential shift. Conversely, in an alkaline environment, deprotonation occurs, resulting in the opposite effect [30,31,32].

Using a three-electrode system with a platinum sheet as the counter electrode and a commercially available Ag/AgCl electrode as the reference electrode, the potential response of the as-prepared pH electrode was tested in an electrolyte solution, with pH values ranging from 3 to 7 over a period of 40 min (Figure 4b). The electrode potential decreased linearly with increasing pH, yielding the linear relationship between the open-circuit potential and pH of *E* = −0.08253 *x* + 0.5924, with a sensitivity of 82.53 mV/pH and a linear correlation coefficient (R^2^) of 0.9973 (Figure 4c). Additionally, the pH electrode demonstrated stability and accuracy across multiple tests in recycling trials (Figure 4d), indicating that the pH electrode prepared in this work is suitable for long-term reuse. Meanwhile, the results of the selectivity test demonstrated that the sensor’s signal remained stable even in the presence of interfering ions such as NH_4_^+^, Na^+^, and Ca^2^^+^ (Figure 4e). This confirms that the electrode can effectively suppress interference from other ions in complex sweat compositions while ensuring the precise detection of the target ion. To further highlight the superior pH-sensing performance of the electrode developed in this work, comparison of the as-prepared electrode with other recently reported PANI-based pH-sensing electrodes was made in terms of the fabrication materials, sensitivity, detection range, and flexibility (Table 1). It can be seen that the pH electrochemical sensing electrode fabricated in this study is superior in its good stretchability and higher sensitivity over other previously reported PANI-based pH-sensing electrodes.

### 3.5. Sensing Stability

**Figure 5:**
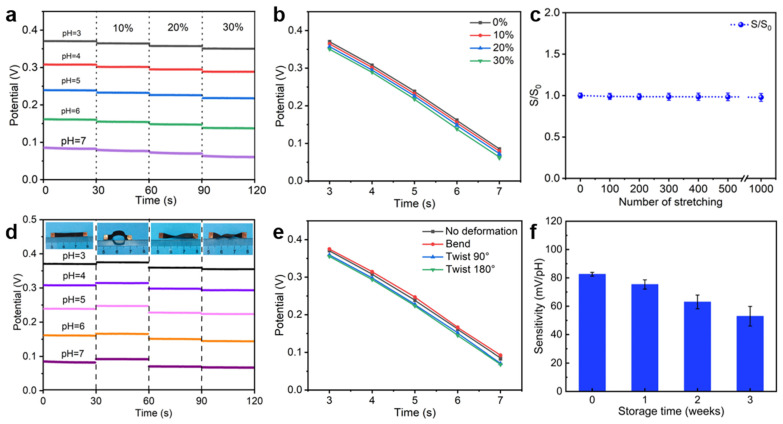
(**a**) Stability of the potential response of pH-sensing electrodes under various tensile deformations and (**b**) the corresponding calibration plots. (**c**) Sensitivity changes in the pH-sensing electrode after 1000 cycles of tensile stretching. (**d**) Stability of the potential response of the pH-sensing electrode under different bending deformations and (**e**) the corresponding calibration plots. (**f**) Changes in the sensitivity of pH-sensing electrodes over storage time.

During human activities, the skin typically stretches in response to joint movements (e.g., up to 30% strain in the arm region) [37]. Consequently, when the sensor is worn on the body, the sensing electrode undergoes deformation along with the movement of the skin and joints. Therefore, it is essential to investigate the electrochemical sensing performance of the electrode under mechanical strain. As shown in Figure 5a, the open-circuit potential response of the electrode was tested in an electrolyte solution with pH value from 3 to 7 at 10%, 20%, and 30% of stretching, respectively. The results showed that the open-circuit potential of the electrode remained stable under different strain levels, as evidenced by the calibration plots in Figure 5b, suggesting that stretching had little impact on the electrode’s sensing performance. Figure 5c demonstrates that the electrode’s sensitivity maintained consistent even after 1000 cycles of repeated stretching, indicating that it can maintain stable sensing performance while providing accurate sweat analysis data during prolonged and frequent human motion. In contrast, the pH-sensing electrode prepared on TPUEM/MWCNT substrate exhibited difficulty in maintaining stable sensing performance even under a 10% tensile strain (Figure S6). Similarly, the open-circuit potential response of the electrode was tested in an electrolyte solution with a pH range of 3–7 under bent, 180° twisted, and 360° twisted states (Figure 5d). The results indicate that the electrode maintains a stable electrical signal output under various deformations. Furthermore, the calibration curves under different deformation conditions (Figure 5e) confirm that torsional deformation has no significant impact on the sensing performance of the electrode. Therefore, these findings demonstrate that the TPUEM/AgNWs/MWCNT-based pH-sensing electrode can function as a stretchable sensor, maintaining effective signal output even during human motion. This excellent stretchability is attributed to the stable conductive pathways within the electrode, which remain intact under deformation, thus ensuring reliable sensing performance.

Figure 5f shows the variation in the sensitivity of the as-prepared pH-sensing electrode with storage time, and it can be seen that the sensitivity of the electrodes was still relatively high for up to three weeks. These results indicate that this pH-sensing electrode based on TPUEM/AgNWs/MWCNT is suitable for use as a stretchable electrode in wearable electrochemical sweat sensors. It is worth noting that with prolonged storage time, the sensitivity of the sensing electrode gradually declines. This degradation is primarily attributed to the partial oxidation of AgNWs within the electrode, leading to a reduction in its electrochemical performance.

## 4. Conclusions

In summary, a stretchable pH-sensing electrode was successfully fabricated by using an electrospun TPU nanofiber membrane as an elastic substrate, sequentially loading AgNWs and MWCNT into the TPU membrane, and then electrochemically depositing PANI via cyclic voltammetry. The as-resulted pH-sensing electrode exhibited a relatively high sensitivity of 82.53 mV/pH, excellent stability upon mechanical deformation, and good reproducibility in ionic solutions with pH ranging from 3 to 7, thus enabling long-term stable real-time pH monitoring. Moreover, in the sensing stability tests, the pH-sensing electrode maintained a stable signal output under various deformations, including stretching (0–30%), twisting, and bending, and retained its original sensing capability even after thousands of repeated stretching cycles, making it suitable for real-world applications in human sweat pH monitoring. However, the long-term storage stability of the electrode could be further improved by incorporating more stable conductive materials. These findings through this study demonstrate that the pH-sensing electrode based on the highly conductive and strain-insensitive TPUEM/AgNWs/MWCNT membrane has strong potential in human sweat pH detection applications.

## Figures and Tables

**Table 1:** Comparison with other recent PANI-based pH electrochemical sensing electrodes.

Materials	Bendable	Stretchable	Sensitivity (mV/pH)	pH Range	Reference
PANI/gold fiber	×	√	60.6	4–8	[20]
PANI/rGO/ITO-PET	×	×	62.3	2–8	[17]
PANI/phytic/interdigital gold electrode	√	×	69.3	4–9	[33]
MoS_2_-PANI/SPCE	√	×	70.4	4–8	[32]
PANI/PVDF yarn	×	×	48.5	4–8	[34]
PANI/H_2_SO_4_/gold layer	×	×	73.4	3–8	[35]
PANI/PP/LIG	×	×	75.1	4–7	[31]
PANI/interdigital electrodes/PI	√	×	58.6	5.5–8.6	[30]
PANI/carbon nanofiber membrane	√	×	76.2	4.0–8.3	[36]
PANI/TPUEM/AgNWs/MWCNT	√	√	82.5	3–7	This work

Abbreviations: rGO, reduced graphene oxide; ITO, indium tin oxide; PET, polyethylene terephthalate; MoS_2_, molybdenum disulfide; SPCE, screen-printed carbon electrode; PVDF, polyvinylidene fluoride; PP, poly (3, 4-ethylene dioxythiophene)-poly (styrene sulfonate) (PEDOT:PSS); LIG, laser-induced graphene; PI, polyimide; “Bendable”, the electrode can maintain stable sensing performance under bending conditions; “Stretchable”, the electrode can maintain stable sensing performance under stretching conditions; “√”, bendable or stretchable; “×”, not bendable or not stretchable.

## Data Availability

The original contributions presented in this study are included in the article/Supplementary Materials. Further inquiries can be directed to the corresponding authors.

## Supplementary Materials

The following supporting information can be downloaded at: https://www.mdpi.com/article/10.3390/polym17050663/s1, Figure S1: SEM image of silver nanowires (AgNWs). Figure S2: (a) EDS mapping results and (b) EDS spectrum and the element percentage of TPUEM/AgNWs/MWCNT. Figure S3: TG curves of TPUEM, TPUEM/AgNWs and TPUEM/AgNWs/MWCNT. Figure S4: Cyclic voltammetry (CV) curves during the electrodeposition of polyaniline on TPUEM/AgNWs/MWCNT electrode. Figure S5: SEM image of the TPUEM/AgNWs/MWCNT electrode surface after cyclic voltammetry scan. Figure S6: (a) Potentiometric response of pH electrode prepared from TPUEM/MWCNT (used as control sample) at 10% strain and (b) its calibration plot.
