# Supplementary material for: Nanofibrous Membrane-Based Stretchable Electrochemical Sweat Sensor for pH Detection
Source: Polymers (Basel). 2025 Feb 28;17(5):663. doi: 10.3390/polym17050663 (PMC11902454; doi:10.3390/polym17050663)
Supplement: Supplementary file 1 [file polymers-17-00663-s001.zip › polymers-3453570-supplementary.pdf]

Supporting Information

Nanofibrous membrane-based stretchable electrochemical sweat sensor for pH detection

Longzhou Zhang [a#], Baoyuan Ma [a#], Zhiguang Xu [b*], Yan Zhao [a*]

[a] College of Textile and Clothing Engineering, Soochow University, Suzhou 215123, China

[b] College of Biological, Chemical Sciences and Engineering, Jiaxing University, Jiaxing 314001, China

*Corresponding authors: zhiguang.xu@zjxu.edu.cn (Z. Xu), yanzhao@suda.edu.cn (Y. Zhao).

[#]These authors contribute equally.

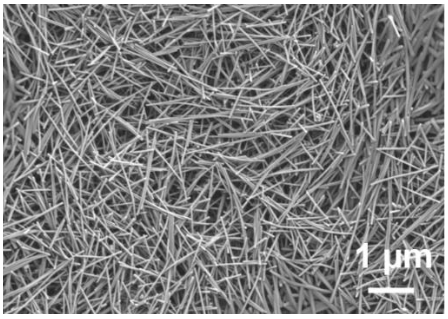

Figure S1 SEM image of silver nanowires (AgNWs).

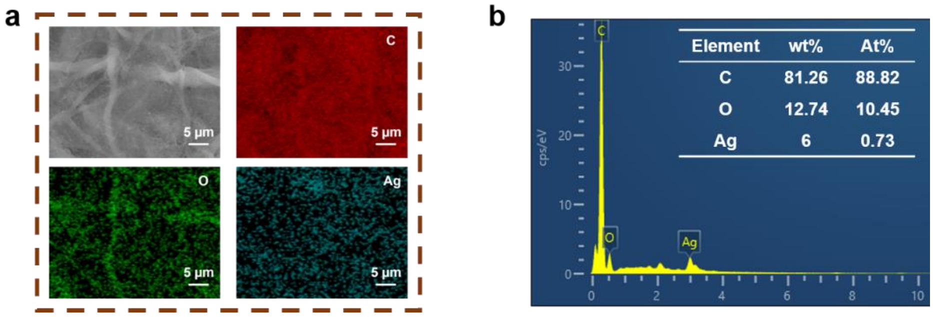

Figure S2 (a) EDS mapping results and (b) EDS spectrum and the element percentage of TPUEM/AgNWs/MWCNT.

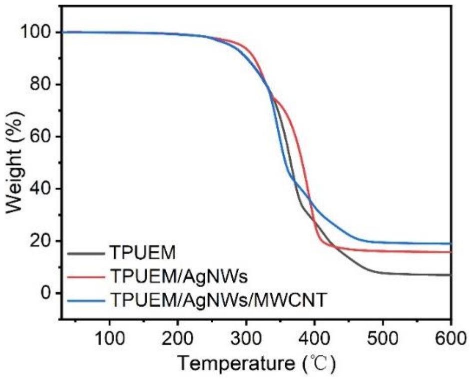

Figure S3 TG curves of TPUEM,TPUEM/AgNWs and TPUEM/AgNWs/MWCNT.

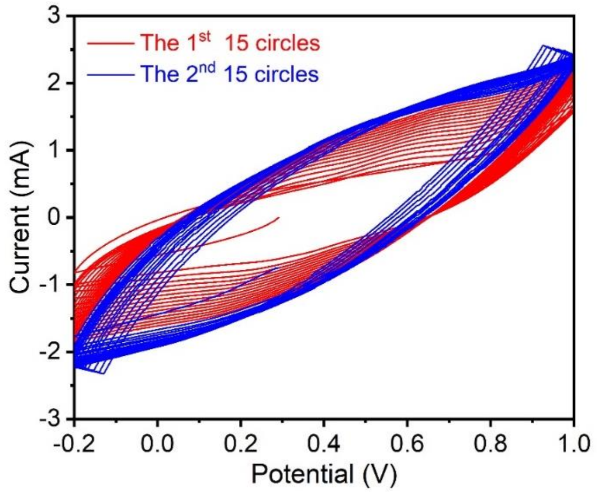

Figure S4 Cyclic voltammetry (CV) curves during the electrodeposition of polyaniline on TPUEM/AgNWs/MWCNT electrode.

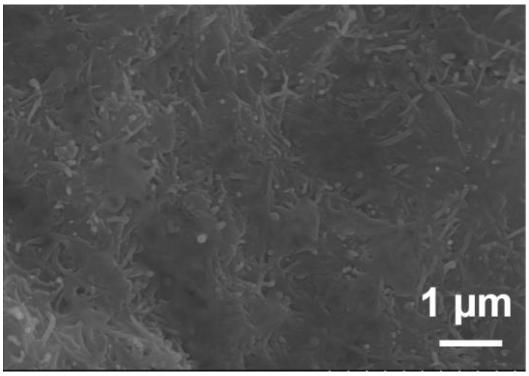

Figure S5 SEM image of the TPUEM/AgNWs/MWCNT electrode surface after cyclic voltammetry scan.

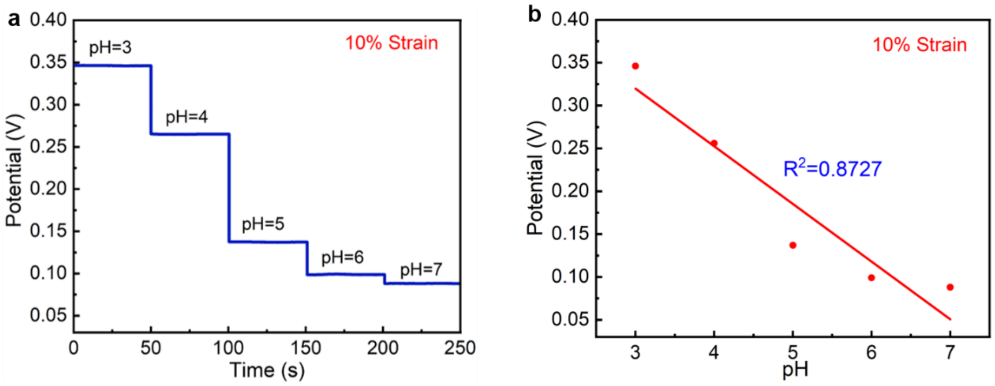

Figure S6 (a) Potentiometric response of pH electrode prepared from TPUEM/MWCNT (used as control sample) at 10% strain and (b) its calibration plot.
